# Clinical determinants of low handgrip strength and its decline in the oldest old: the Leiden 85-plus Study

**DOI:** 10.1007/s40520-020-01639-4

**Published:** 2020-06-30

**Authors:** Carolina H. Y. Ling, Jacobijn Gussekloo, Stella Trompet, Carel G. M. Meskers, Andrea B. Maier

**Affiliations:** 1 Internal Medicine Department, The Prince Charles Hospital, Chermside, QLD Australia; 2 Department of Gerontology and Geriatrics, Leiden University Medical Center, Leiden, The Netherlands; 3 Department of Human Movement Sciences, Faculty of Behavioural and Movement Sciences, VU University Amsterdam, Amsterdam Movement Sciences, Amsterdam, The Netherlands; 4 Department of Rehabilitation Medicine, Amsterdam UMC, VU University Medical Center, Amsterdam Movement Sciences, de Boelelaan 1118, 1081 HZ Amsterdam, The Netherlands; 5 Department of Medicine and Aged Care,, The Royal Melbourne Hospital, University of Melbourne, Melbourne, Australia

**Keywords:** Muscle strength, Sex differences, Physical fitness, Cognitive function

## Abstract

**Background:**

Age-related decline in muscle strength, dynapenia, is linked to serious adverse health outcomes. Evidence on the determinants of muscle strength decline in the oldest old is lacking.

**Aims:**

To identify clinical variables associated with handgrip strength and its change over a 4-year period in an oldest old cohort.

**Methods:**

We included 555 participants from the Leiden 85-plus Study, a prospective population-based study of 85-year-old inhabitants of Leiden, the Netherlands. Handgrip strength was assessed at age 85 and 89 years. Anthropometry, mental status, functional performance, and biochemical variables were obtained at baselines. Significant univariates were included into multivariable regression models to extract the final predictive variables.

**Results:**

Handgrip strength for men and women at age 85 years was 30.6 kg (SD 8.2) and 18.7 kg (SD, 5.5), respectively. In the cross-sectional analysis, body height and weight were positively associated with handgrip strength in both genders. Higher functional performance was associated with stronger handgrip strength in women. Mean absolute handgrip strength decline over 4 years was greater for men than women (− 6.1 kg (SD, 5.2) vs. − 3.4 kg (SD, 4.1), *p* < 0.001). Men with better baseline cognitive functioning had smaller decline in handgrip strength.

**Conclusions:**

This study further strengthens evidence linking functional and cognitive performances to muscle strength in the oldest old. Future research is needed to ascertain causality and determine if these markers represent potential targets for intervention.

## Introduction

Low muscle strength, also known as dynapenia [1], is highly prevalent, with reported rates for community dwelling male and female adults estimated to be 19% and 27%, respectively, for those aged 60 to 69 years, 31% and 42% for those aged 70 to 79 years, and around 45% for those aged 80 years and above [2]. Low muscle strength is associated with various adverse health outcomes including increased falls [3], functional and cognitive decline [4], and all cause mortality [5]. The negative effects of low muscle strength are most profound in the older adults. Pathophysiological process underlying muscle strength loss with age is rather complex and comprises of a series of interplay between genetic, environmental, and lifestyle factors which are not yet been fully elucidated [6, 7].

Several factors including low body weight [8], chronic diseases such as diabetes [9] and chronic obstructive pulmonary disease [10], and physical inactivity [11] have been shown to be associated with poor muscular strength in the middle-aged and older adults. However, evidence on the determinants of low muscle strength and its decline in the oldest old is lacking. Age-related physiological changes and the increased prevalence of multimorbidity in this age group may affect relationships between clinical variables and muscle strength.

Addressing the abovementioned relationships requires longitudinal studies in the oldest old. This may provide greater insight into the pathogenesis of muscle strength decline in this particularly vulnerable population and may guide future development of preventive and therapeutic strategies. Therefore, the current study aimed to identify clinical variables associated with handgrip strength and its change over a period of four years in a cohort of oldest old individuals.

## Methods

### Study design and participants

Data were obtained from the Leiden 85-plus Study, a prospective population-based study of 85-year-old inhabitants living in the city of Leiden in The Netherlands. Between September 1997 and September 1999, all adults born between the year from 1912 to 1914 who turned 85 years of age were invited to participate. 599 subjects were enrolled in the study (response rate 87%). There were no selection criteria on health or demographic characteristics. Subjects were visited annually in their homes where face-to-face medical interviews, physical examination, blood samples, and various mental and physical function tests including handgrip strength were performed. Further details on the design of the study and characteristics of the cohort have been published elsewhere [12]. The Medical Ethical Committee of the Leiden University Medical Center approved the study, and informed consent was obtained from all the subjects.

### Clinical variables

Basic demographics (gender and living arrangement) and medical comorbidities such as cardiovascular disease (i.e., ischemic heart disease, cerebrovascular disease and peripheral vascular disease), diabetes mellitus, chronic obstructive pulmonary disease, neoplasm, Parkinson’s disease and arthritis (i.e., osteoarthritis, rheumatoid arthritis and polymyalgia rheumatica)], presence of polypharmacy (defined as five or more regular medications), and smoking status were extracted from the medical interviews. Blood pressure and anthropometric measurements including height and weight were obtained from the clinical examination data. Body mass index (BMI, kg/m^2^) was calculated as weight (kg)/height(m)^2^ and body surface area (BSA, m^2^) was calculated using the Mosteller formula: √(height (cm) × weight (kg)/3600) [13]. Mental performance was assessed by means of the Mini-Mental State Examination (MMSE, score range 0–30 points) and Geriatric Depression Scale (GDS, score range 0–15 points). Functional performance was assessed using the Groningen Activity Restriction Scale (GARS, score range 18 (not disabled) to 72 (severely disabled)) [14] and physical activity score (PAS, comprised of four items from the Time Spending Pattern questionnaire, score range 0–16)) [15]. Biochemical variables including serum haemoglobin, creatinine, albumin, and C-reactive protein (CRP) were selected as potential variables associated with handgrip strength.

### Outcome measure

Handgrip strength measurements were available in 555 of the 599 participants enrolled in the Leiden 85-plus study at age 85 years. Of the 44 participants without completed handgrip strength measurements, 17 were due to physical impairment, 9 due to cognitive impairment, 5 due to inability to follow instructions, 3 due to refusal to participate, and 10 due to other reason. The measurements for the dominant hand were obtained (to the nearest kilograms) using a Jamar hand dynamometer (Sammons Preston Inc., Bolingbrook, IL), with the participant in an upright position and the arm unsupported and parallel to the body. The width of the dynamometer handle was adjusted to the participant’s hand size such that the middle phalanges rested on the inner handle. The participant was advised to exert maximal force and one test trial was allowed, followed by three test measurements [16]. The highest measurement recorded was used in the final analysis. Dynapenia was defined as handgrip strength < 30 kg for men and < 20 kg for women [17].

Three hundred and fifty-seven participants were alive at follow-up age 89 years and handgrip strength measurements were available in 304 (85%) of them. Participants without handgrip strength at follow-up were not significantly different from participants with handgrip strength at follow-up in terms of comorbidities, cognitive, and functional performances at baseline [5].

### Statistical analysis

Continuous variables with Gaussian and non-Gaussian distributions are presented as mean (standard deviation, SD) and median (interquartile range, IQR) respectively. Non-linear independent variables were categorized into quartiles or log transformed. All data were analyzed separately for women and men. Univariable linear regression was used to determine the cross-sectional associations between the baseline clinical variables and handgrip strength at age 85 years. Significant clinical variables (*p* < 0.10) were entered into a multivariable linear regression model using the simultaneous method. Likewise, in the longitudinal analysis, the association between baseline clinical variables and absolute change in handgrip strength (handgrip strength at age 89 years minus handgrip strength at age 85 years) was first examined using univariable linear regression, adjusted for baseline handgrip strength. Significant univariates (*p* < 0.10) were entered into a multivariable linear regression model using the simultaneous method. Multicollinearity between independent variables was assessed by examining the tolerance level generated from the multivariable regression analysis. Variables with tolerance level ≤ 0.40 were excluded from the model. A 2-tailed *p* < 0.05 was considered significant. All statistical analyses were performed using SPSS for Windows (SPSS Inc, Chicago), version 16.

## Results

### Subjects characteristics

Clinical characteristics of the subjects according to gender at baseline age 85 years are presented in Table 1. Both genders were similar in their disease burden, with cardiovascular disease being the most common comorbidity. The mean handgrip strength for men and women at baseline were 30.6 kg (SD 8.2) and. 18.7 kg (SD, 5.5), respectively. The prevalence rates for dynapenia were 42.3% and 49.3% for men and women, respectively.

**Table 1:** Characteristics at baseline age 85 years of the participants of the Leiden 85-plus Study, according to gender

Clinical variables	Women (*N* = 361)	Men (*N* = 194)
Living arrangement (%)		
Institutionalization	15.9	8.9
Comorbidity (%)		
Cardiovascular disease^a^	59.8	67.5
Diabetes mellitus	16.6	12.9
COPD	8.9	17.3
Neoplastic disease	15.0	23.4
Parkinson’s disease	1.4	3.1
Arthritis^b^	35.6	27.5
Comorbidity sum score^c^, median (IQR)	1 (1–2)	1 (1–2)
Medications (%)		
Polypharmacy^d^	19.8	15.3
Smoking status (%)		
Current smoker	9.1	27.5
Non-smoker/ex-smoker	90.9	72.5
Anthropometry, mean (SD)		
Height, cm	156 (6)	168 (6)
Weight, kg	67.7 (12.7)	74.1 (11.9)
BMI, kg/m^2^	27.7 (4.8)	26.1 (3.7)
BSA, m^2^	1.71 (0.18)	1.86 (0.17)
Blood pressure, mmHg		
Systolic	156 (18)	156 (20)
Diastolic	78 (9)	76 (10)
Mental performance, median (IQR)		
MMSE	26 (22–28)	26 (24–28)
GDS-15	2 (1–3)	2 (1–3)
Functional performance, median (IQR)		
GARS	28 (21–39)	26 (21–36)
PAS	6.0 (4.0–8.0)	7.0 (5.0–9.0)
Biochemical parameters, mean (SD)		
Haemoglobin, g/L	129 (12)	134 (15)
Creatinine, umol/L	90.3 (20.1)	115.3 (46.4)
Albumin, g/L	41.9 (2.9)	42.1 (3.4)
CRP, mg/L, median (IQR)	3 (1–7)	4 (2–8)
Handgrip strength, kg	18.7 (5.5)	30.6 (8.2)

*BMI* body mass index, *BSA* body surface area, *MMSE* mini-mental state examination (score range 0–30), *GDS-15* Geriatric Depression Scale-15 (score range 0–15), *GARS* Groningen Activity Restriction Scale (score range 18–72), *PAS* physical activity score (sum score of 4 physically active items from the Time Spending Pattern questionnaire, score range 0–16), *SD* standard deviation, *IQR* interquartile range

^a^Cardiovascular disease includes ischemic heart disease, cerebrovascular disease, and peripheral vascular disease

^b^Arthritis includes osteoarthritis, rheumatoid arthritis, and polymyalgia rheumatica

^c^Comorbidity sum score represents the total sum of the six comorbidities

^d^Polypharmacy =  ≥ 5 regular medications

### Clinical variables associated with handgrip strength at age 85 years

Table 2 shows the results of the multivariable regression analysis for associations between baseline clinical variables and handgrip strength at age 85 years according to gender. Height and weight were positively associated with handgrip strength in both genders. Lower GARS score was associated with stronger handgrip strength in women (*p* < 0.001). A positive association was observed between systolic blood pressure and handgrip strength in men, but this did not reach statistical significance (*p* = 0.053).

**Table 2:** Associations between baseline clinical variables and handgrip strength at age 85 years according to gender (multivariable linear regression)

Clinical variables	Handgrip strength, kg			
	Women (*N* = 361)		Men (*N* = 194)	
	Unstandardized B (SE)	*p*-value	Unstandardized B (SE)	*p*-value
Institutionalization	− 1.59 (0.89)	0.075	− 3.31 (2.34)	0.160
Cardiovascular disease			− 0.31 (1.39)	0.822
Comorbidity sum score			− 0.73 (0.67)	0.277
Height, cm	0.13 (0.05)	0.006	0.36 (0.11)	0.001
Weight, kg	0.09 (0.02)	< 0.001	0.13 (0.06)	0.021
Systolic blood pressure, mmHg	0.03 (0.02)	0.180	0.07 (0.04)	0.053
Diastolic blood pressure, mmHg	− 0.02 (0.04)	0.671	− 0.03 (0.07)	0.738
MMSE^a^	0.19 (0.29)	0.521	− 0.18 (0.61)	0.768
GDS-15^a^	− 0.35 (0.27)	0.201	− 0.30 (0.57)	0.596
GARS^a^	− 1.08 (0.28)	< 0.001	− 0.41 (0.62)	0.509
PAS^a^	0.29 (0.26)	0.269	0.70 (0.56)	0.208
Haemoglobin, g/L	− 0.02 (0.03)	0.486		
Albumin, g/L	0.12 (0.11)	0.252	0.00 (0.18)	0.995
CRP, mg/L^a^			− 0.69 (0.53)	0.189

*R*^2^ (women) = 0.253; *R*^2^ (men) = 0.305; BMI and BSA variables were removed from the multivariable linear regression model due to multicollinearity

^a^Variable was divided into quartiles

### Baseline clinical variables associated with absolute change in handgrip strength from age 85 to 89 years, adjusted for baseline handgrip strength

At follow-up age 89 years, the mean values of handgrip strength for men and women were 25.6 kg (SD 7.8) and 16.4 kg (SD 5.0), respectively. The absolute handgrip strength decline from age 85 to 89 years was greater for men than women (− 6.1 kg (SD, 5.2) vs. − 3.4 kg (SD, 4.1), *p* < 0.001).

Table 3 shows the results of the multivariable regression analysis for associations between baseline clinical variables and absolute change in handgrip strength from age 85 to 89 years, adjusted for baseline handgrip strength, according to gender. Higher baseline MMSE score was associated with less handgrip strength decline in men (*p* = 0.044). No significant associations were demonstrated between the clinical variables and absolute handgrip strength decline in women.

**Table 3:** Associations between baseline clinical variables and absolute change in handgrip strength from age 85 to 89 years, adjusted for baseline handgrip strength, according to gender (multivariable linear regression)

Clinical variables	Absolute change in handgrip strength, kg			
	Women (*N* = 215)		Men (*N* = 89)	
	Unstandardized B (SE)	*p*-value	Unstandardized B (SE)	*p*-value
Cardiovascular disease	− 0.65 (0.53)	0.222		
Non-smoker/ex-smoker			1.72 (1.24)	0.169
Height, cm	0.07 (0.04)	0.125		
MMSE^a^	0.47 (0.28)	0.096	1.15 (0.56)	0.043
GDS-15^a^	− 0.39 (0.25)	0.129		
GARS^a^	− 0.40 (0.29)	0.166	− 0.48 (0.52)	0.354
CRP, mg/L^a^			− 0.67 (0.49)	0.172

*R*^2^ (women) = 0.213; *R*^2^ (men) = 0.230; current smoker variable was removed from the multivariable linear regression model due to multicollinearity

^a^Variable was divided into quartiles

## Discussion

In a cohort of oldest old, clinical determinants of handgrip strength at age 85 years and its decline over a 4-year period were different for men and women, with the exception of height and weight. In women, a positive cross-sectional association was observed between baseline functional performance and handgrip strength. Better baseline cognitive performance was associated with less handgrip strength decline in men.

Handgrip strength values were higher in men compared to women, consistent with previous reported normative data for older adults [18, 19]. Sixty-four percent of our study participants were alive at 89 years of age, which was comparable to survival rate in the Newcastle 85+ Study [20]. A steeper handgrip strength decline was observed in men during the follow-up period, in line with the previous literature [21, 22]. The high prevalence rate of dynapenia in our oldest old cohort was in concordance with earlier reports [2, 23].

The finding of positive relationships between height and weight with handgrip strength in both genders are not unexpected and consistent with previous reports [24, 25]. Of the anthropometric parameters, height has been shown to be most significantly correlated with handgrip strength [26]. It is postulated that height reflects bone structure and mass, which in turn has implications on muscle strength and performance [24]. Taller individuals also generally have longer limbs and greater lever arm resulting in more efficient force generation [27]. Several studies have also demonstrated the association between body weight and muscle strength [25]. For adults with healthy weight range, body weight correlated significantly with lean body mass [28], which strongly predicts upper body strength [29].

A positive cross-sectional association was demonstrated between functional performance and handgrip strength, but this was observed in women only. It is possible that women may have overreported or men underreported their disabilities. Furthermore, no association was seen between baseline functional performance and absolute handgrip strength decline in either gender, contrary to results of previous research [30]. This discrepancy may be explained by the self-reported nature of the Groningen Activity Restriction Scale used to measure functional performance in this study. Moreover, functional performance of the participants might have been enhanced by external factors such as established infrastructure aimed at preserving functional capacity and the use of coping strategies and adaptation. There is clear evidence that physical function and muscle strength are linked. Weak muscular strength contributes to physical limitation and functional impairment, which in turn sets up a vicious cycle for increased dependence and deconditioning with subsequent muscle strength loss [4, 31, 32]. Our finding of a lack of temporal association between baseline functional performance and absolute handgrip strength decline suggests that the weak handgrip strength may be the inciting event in the relationship.

Although not reaching statistical significance, it is worth noting the positive association observed between systolic blood pressure and handgrip strength in men. Analysis of the National Health and Nutrition Examination Survey (NHANES) database [33] showed that after adjusting for BMI, handgrip strength was positively associated with both systolic and diastolic blood pressure in men but only with diastolic blood pressure in women. The participants in our study were older and had overall higher mean systolic and diastolic blood pressure, and therefore, the results may not be comparable to that of NHANES. Aging contributes to a reduction in arterial compliance and increased peripheral vascular resistance leading to a rise in systolic blood pressure. It has been suggested that higher systolic blood pressure in the older adults may be an essential compensatory mechanism to maintain perfusion and prevent tissue injury of major end organs such as the brain, kidney, and muscle tissue [34]. This may explain the relationship between higher systolic blood pressure and better muscle function in older adults.

Better baseline cognitive performance was associated with less absolute handgrip strength loss from age 85 to 89 years in men. Cognitive dysfunction has been shown to be associated with lower level of physical activity [35] and dietary insufficiency [36], both of which contribute to loss of muscle mass and strength. Several possible common underlying mechanisms such as oxidative stress and high inflammatory markers may account for the concomitant loss of brain cells and motor neurons leading to muscle loss and weakness in people with cognitive impairment [37].

The present study has several notable strengths. The Leiden 85-plus Study is a population-based cohort study of the oldest old with comprehensive health and functional measures. The oldest old are often excluded from research, and therefore, there is a paucity of direct evidence to guide interventions in this population. Being the fastest growing sector, it is essential for up to date data to be available to allow better management and future planning. The longitudinal design and repeated clinical measures of our study enable us to study temporal associations. This study also has several limitations. Firstly, the assessments of comorbidities were limited to common chronic diseases and we were not able to adjust for the interim development of new comorbidities or account for the disease severity. Secondly, the number of men were lower compared to women*;* it cannot be excluded that this led to inadequate power in men to identify clinical variables associated with handgrip strength. Of note, this study was conducted in a homogenous Dutch population, and therefore, the findings might not be generalizable to older populations of another ethnicity.

## Conclusion

This study further strengthens support for the associations between functional and cognitive performances with muscle strength in the oldest old. Given the lack of association of other clinical determinants with change of handgrip strength and the predictive validity of handgrip strength for multiple important health outcomes, it emphasizes the importance of routine handgrip strength measurements in older adults. Future research is needed to establish causal relationships and determine whether these markers represent potential targets for intervention.

## Data Availability

All authors had full access to the study data (including statistical reports and tables) and can take responsibility for the data integrity and the accuracy of the analysis.
